# Intrinsic chaos control in cortical circuits: A minimal E-I-M rate model for primary visual cortex

**DOI:** 10.1007/s10827-026-00938-5

**Published:** 2026-06-22

**Authors:** Mehdi Borjkhani, Morteza A. Sharif, Hadi Borjkhani

**Affiliations:** 1 International Centre for Translational Eye Research (ICTER), Institute of Physical Chemistry, Polish Academy of Sciences, Warsaw, Poland; 2 Optics and Laser Engineering Group, Faculty of Electrical Engineering, Urmia University of Technology, Urmia, Iran; 3 Hochschule für Technik und Wirtschaft (HTW) Berlin, Berlin, Germany

**Keywords:** Neural population dynamics, Chaos control, E-I balance, Visual cortex, Rate model, Homeostatic regulation, Orientation selectivity

## Abstract

Cortical circuits exhibit variable yet bounded activity patterns, suggesting operation near—but not within—fully chaotic regimes. Here we develop a minimal three-variable rate model for primary visual cortex (V1) that reveals how biologically motivated feedback mechanisms can function as intrinsic chaos controllers. We adopt the simplest known chaotic Lotka–Volterra system as a phenomenological scaffold and introduce three biologically motivated modifications: excitatory-to-inhibitory (E*→ *I) feedback coupling, homeostatic regulation of modulatory drive, and orientation-tuned sensory input. These modifications transform the excitatory (E), inhibitory (I), and modulatory (M) population dynamics from chaotic strange attractors into controlled limit cycles—a 93% reduction in dynamical variance. The model reproduces key V1 phenomena: orientation selectivity matching experimental distributions (OSI *= 0.38 ± 0.09*), stimulus-induced variability quenching, and realistic spiking irregularity when coupled to Hodgkin–Huxley neurons (CV$_{\text {ISI}} = 0.27$, within *in vitro* range). Parameter space analysis reveals that feedback mechanisms robustly stabilize activity across most of the tested chaotic regime. We further demonstrate that, within this minimal structure, the specific $(1-\textrm{I}^2)$ disinhibition nonlinearity enables chaos—bounded alternatives tested do not support chaotic dynamics. Our findings suggest that cortical circuits possess an intrinsic capacity for chaos that is actively suppressed by canonical feedback motifs, positioning the brain at the edge of instability where computational flexibility meets reliable signal processing.

## Introduction

### The variability paradox in cortical processing

Cortical neurons face a fundamental challenge: they must respond reliably to sensory inputs while maintaining the flexibility required for adaptive computation. Single-unit recordings reveal highly irregular firing patterns with coefficients of variation (CV) approaching unity (Shadlen & Newsome, 1998; Softky & Koch, 1993), yet stimulus-evoked responses are remarkably consistent across trials once variability is properly accounted for Churchland et al. (2010). This “variability paradox” suggests that cortical circuits operate in a dynamical regime that balances irregularity with stability.

Deterministic chaos offers a compelling framework for understanding this balance. Chaotic systems generate complex, aperiodic dynamics from simple deterministic rules, producing apparent randomness without requiring explicit noise sources (van Vreeswijk & Sompolinsky, 1996). However, pure chaos would render neural coding unreliable—small perturbations would catastrophically diverge, preventing consistent stimulus representations. The question thus becomes: how do cortical circuits harness the computational benefits of near-chaotic dynamics while avoiding pathological instability?

### Minimal models and the search for essential mechanisms

Population rate models have proven invaluable for isolating essential mechanisms in neural dynamics. The Wilson–Cowan equations (Wilson & Cowan, 1972, 1973) established that excitatory-inhibitory (E-I) interactions alone can generate rich dynamics including oscillations and bistability. However, as two-dimensional systems, they cannot exhibit chaos due to the Poincaré–Bendixson theorem. Extending to three dimensions permits chaotic dynamics, but identifying the *minimal* three-variable system capable of generating biologically plausible E-I chaos has remained elusive.

Recently, Jafari et al. (2025) discovered the simplest known chaotic Lotka–Volterra (LV) system—containing only seven terms and two parameters. This mathematical parsimony is attractive for theoretical neuroscience, where minimal models help distinguish necessary from sufficient mechanisms. However, direct neural interpretation of this system raises immediate concerns: the equations lack standard features of cortical circuits, including feedback from excitatory to inhibitory populations and homeostatic regulation of activity.

### Relation to prior models of cortical chaos

The question of whether and how cortical circuits generate chaotic dynamics has been a central one in theoretical neuroscience for nearly four decades. A first major thread, originating with the seminal work of Sompolinsky et al. (1988), established that random recurrent networks of $N\rightarrow \infty$ rate units undergo a sharp transition to high-dimensional chaos as synaptic gain crosses a critical value. van Vreeswijk and Sompolinsky (1996, 1998) showed that chaotic asynchronous activity emerges generically in balanced networks of excitatory and inhibitory spiking neurons, providing a foundational explanation for irregular cortical firing. Subsequent work refined this picture along multiple axes: Brunel (2000) mapped the phase diagram of sparse spiking networks; Renart et al. (2010) clarified how tracking inhibition produces the asynchronous state; Monteforte and Wolf (2010) characterized the microscopic structure of the chaotic attractor; Ostojic (2014) demonstrated a transition between rate-stable and rate-fluctuating asynchronous regimes; and Kadmon and Sompolinsky (2015) and Mastrogiuseppe and Ostojic (2017) extended these analyses to networks with biologically constrained transfer functions and segregated excitatory–inhibitory subpopulations. A complementary line of work has examined how external stimuli interact with intrinsic chaos: Rajan et al. (2010) showed that input drives high-dimensional networks through a phase transition that suppresses ongoing chaos, and Lajoie et al. (2016) characterized how this stimulus-driven suppression shapes encoding.

While most of this literature concerns high-dimensional networks, recent work has investigated low-dimensional chaotic regimes more directly relevant to the present study. Wilson (2019) demonstrated that small grids of coupled Wilson–Cowan oscillators generate hyperchaos with a complexity that scales linearly with system size, and discussed implications for unpredictability in human visuomotor sequences. Mosheiff et al. (2023) showed that spatially distributed E-I networks with matched projection widths produce low-dimensional chaotic patterns consistent with the shared variability observed in cortical recordings. Xie et al. (2025) characterized a slow, broadened transition to low-dimensional chaos in finite recurrent networks with heavy-tailed connectivity. Particularly relevant to our work, Nicola et al. (2018) demonstrated that homeostatic regulation operating on a slow timescale can *generate* chaos in Wilson–Cowan systems—a finding that is, in a sense, the dynamical inverse of what we report below.

The present work occupies a distinct niche in this landscape. Rather than asking how chaos *emerges* in large or random networks, we ask the complementary question: *what is the minimal three-dimensional E-I-M circuit that supports chaos, and what is the smallest feedback structure that controls it?* Working in this deliberately minimal regime affords two things. First, it makes the chaos-control mechanism analytically tractable in a way that high-dimensional analyses, which rely on mean-field statistics, cannot easily achieve for individual feedback motifs. Second, it generates predictions specific to the low-dimensional regime—about the relative contributions of E*→ *I coupling and homeostatic timescale, about the role of disinhibitory motifs, and about how stimulus-driven attractor collapse may complement the high-dimensional suppression mechanism of Rajan et al. (2010)—that we develop in Sections 4 and 5. We do not claim that our minimal model supersedes the high-dimensional balanced-network framework; rather, we view the two scales as complementary descriptions of how cortex navigates between order and chaos.

### Present contribution

We show that introducing biologically motivated constraints into the minimal chaotic LV system fundamentally transforms its dynamics. Specifically, we demonstrate that:

1. E*→ *I feedback coupling and homeostatic regulation convert chaotic attractors into stable limit cycles
2. This “chaos control” emerges from canonical cortical circuit motifs rather than parameter fine-tuning
3. The controlled system reproduces key experimental phenomena including orientation selectivity, variability quenching, and realistic spiking statistics
4. The specific $(1-\textrm{I}^2)$ disinhibition nonlinearity enables chaos within this minimal structure—bounded alternatives tested do not support chaotic dynamics

These findings suggest that cortical circuits possess an intrinsic capacity for chaos that is actively suppressed by feedback mechanisms, positioning neural dynamics at the computationally advantageous edge of instability.

## The E-I-M model

### From ecology to neuroscience: A phenomenological mapping

The Jafari et al. chaotic LV system describes three interacting populations:

1 $$\begin{aligned} \dot{x}&= -x(1 - y^2) \nonumber \\ \dot{y}&= -y(ay^2 - z^2) \\ \dot{z}&= z(b - x^2 - y^2) \nonumber \end{aligned}$$

where *a* and *b* are control parameters. We assign these variables qualitative neural roles consistent with the canonical V1 microcircuit:

2 $$\begin{aligned} \textrm{E}(t)&\equiv x(t) \quad \text {: Excitatory pyramidal neurons} \nonumber \\ \textrm{I}(t)&\equiv y(t) \quad \text {: Fast inhibitory interneurons (PV+)} \\ \textrm{M}(t)&\equiv z(t) \quad \text {: Modulatory drive (thalamic + neuromodulatory)} \nonumber \end{aligned}$$

This assignment is consistent with the dominant cell-class composition of V1: pyramidal cells comprise *∼ *80% of cortical neurons and provide glutamatergic excitation; parvalbumin-positive (PV+) interneurons mediate fast GABAergic inhibition with strong coupling to pyramidal output (Atallah & Scanziani, 2009; Markram et al., 2004); and thalamocortical/neuromodulatory systems provide slower external drive (Harris & Thiele, 2011).

#### Interpretive scope of the mapping

We emphasize that the LV system is adopted here as a *phenomenological dynamical-systems scaffold*, not as a model derived from synaptic biophysics or from formal mean-field analyses of spiking networks (e.g., Bressloff, 2010; Buice & Cowan, 2007; Ermentrout & Terman, 2010; Sompolinsky et al., 1988; van Vreeswijk & Sompolinsky, 1996; Wilson & Cowan, 1972; 1973). In particular, the polynomial interaction terms appearing in Eq. (1)—such as $\textrm{E}\textrm{I}^2$, $a\textrm{I}^3$, and $\textrm{I}\textrm{M}^2$—do not arise from any standard mean-field reduction and should not be read as direct correlates of specific synaptic mechanisms (e.g. a cubic term does not represent a literal three-spike interaction). Our use of this system is methodological: among three-dimensional polynomial systems whose three variables admit any qualitative neural interpretation, the Jafari et al. equations represent the simplest known chaotic example. We exploit this minimality to ask a focused mechanistic question—*what is the smallest perturbation, in the form of canonical cortical feedback motifs, that converts a chaotic 3D rate system into a controlled one?* Accordingly, the model parameters *a*, *b*, $k_\textrm{I}$, *γ * should be understood as control knobs governing E–I balance, background drive, feedback strength, and homeostatic timescale, rather than as direct correlates of conductances or receptor densities. We discuss the trade-offs of this minimal formulation against more biophysically detailed alternatives in Section 4.4.4, and we summarize the resulting predictions in Section 5.

### Augmenting the system for cortical plausibility

Even granting the phenomenological framing of Section 2.1, three features of the bare system in Eq. (1) are at odds with well-established cortical phenomenology and motivate targeted augmentation:

#### Issue 1: Missing E*→ *I drive

The $\textrm{I}$ equation contains no excitatory input, contradicting the strong pyramidal*→ *PV drive observed experimentally (Atallah & Scanziani, 2009; Pfeffer et al., 2013). Any model that aspires to even loose correspondence with E–I cortical circuitry should include this coupling.

#### Issue 2: Unstable modulatory dynamics in the silent regime

When $\textrm{E}= \textrm{I}= 0$, we have $\dot{\textrm{M}} = b\textrm{M}$, yielding unbounded exponential growth for *b > 0*. Real neuromodulatory systems are homeostatically regulated (Turrigiano & Nelson, 2004); we therefore add a leak term that pulls $\textrm{M}$ toward a baseline.

#### Issue 3: Sign reversal of the inhibitory factor

The term $(1 - \textrm{I}^2)$ becomes negative when $|\textrm{I}| > 1$, effectively converting net inhibition to net excitation in Eq. (1). This is mathematically required for chaos in the bare system but warrants interpretive care, which we provide in Section 2.4.

These three considerations motivate the augmentations introduced below. We do not claim that the augmented terms “correct” the LV system in any biophysical sense—rather, they bring its qualitative behavior into closer alignment with established cortical phenomenology while preserving the minimal polynomial structure that makes chaos analytically tractable.

### The augmented E-I-M system

We introduce three modifications addressing these limitations:

3 $$\begin{aligned} \dot{\textrm{E}}&= -\textrm{E}(1 - \textrm{I}^2) + k_\textrm{E}\cdot S(t; \theta ) \end{aligned}$$

4 $$\begin{aligned} \dot{\textrm{I}}&= -\textrm{I}(a\textrm{I}^2 - \textrm{M}^2) + k_\textrm{I}\cdot \textrm{E} \end{aligned}$$

5 $$\begin{aligned} \dot{\textrm{M}}&= \textrm{M}(b - \textrm{E}^2 - \textrm{I}^2) - \gamma (\textrm{M}- \textrm{M}_0) \end{aligned}$$

The new terms are:

- $+k_\textrm{I}\cdot \textrm{E}$: E*→ *I feedback implementing pyramidal*→ *PV drive
- $-\gamma (\textrm{M}- \textrm{M}_0)$: Homeostatic regulation maintaining $\textrm{M}$ near baseline $\textrm{M}_0$
- $+k_\textrm{E}\cdot S(t; \theta )$: Orientation-tuned visual stimulus input

Table 1 summarizes parameter interpretations and experimental correlates.

**Table 1:** Parameter interpretations and experimental manipulations

Param.	Functional Role	Cortical Correlate	Experimental Manipulation
*a*	Inhibitory nonlinearity	PV interneuron gain	GABAergic drugs, PV optogenetics
*b*	Background drive	Thalamic/arousal tone	Anesthesia, attention tasks
$k_\textrm{E}$	Stimulus coupling	LGN*→ *V1 strength	Visual contrast, thalamic silencing
$k_\textrm{I}$	E-I feedback	Pyr*→ *PV synapse	Glutamate receptor antagonists
*γ *	Homeostatic rate	Synaptic scaling	Chronic activity manipulation
$\textrm{M}_0$	Baseline drive	Resting thalamic activity	Baseline arousal state

#### Timescales and dimensional conventions

Equations (3)–(5) are written in dimensionless form: time is normalized so that the implicit decay timescale of each population is unity ($\tau _\textrm{E}= \tau _\textrm{I}= \tau _\textrm{M}= 1$), and population activities are dimensionless rates rather than spikes per second. Differences between the biophysical timescales of pyramidal cells (membrane time constant *∼ *10–20 ms), PV interneurons (*∼ *5–10 ms), and slow modulatory drive (*∼ *100–1000 ms) are therefore not represented explicitly in this minimal formulation. The homeostatic rate *γ * does, however, introduce a second effective timescale by determining how quickly $\textrm{M}$ relaxes toward $\textrm{M}_0$: small *γ * corresponds to slow homeostatic adaptation relative to the E–I dynamics, while large *γ * produces fast modulatory equilibration. Multi-timescale extensions in which $\tau _\textrm{E}, \tau _\textrm{I}, \tau _\textrm{M}$ are allowed to differ would be a natural next step but are deferred here in keeping with the minimal-model framing of Section 2.1. Crucially, the chaos-control mechanism we identify does not rely on the absolute values of these timescales—only on the existence of a homeostatic relaxation rate *γ * that is non-zero relative to the intrinsic E-I-M dynamics. The signs of the population variables, rather than their absolute biophysical timescales, distinguish $\textrm{E}$ from $\textrm{I}$ at the level of their effect on each other in Eqs. (3)–(4): $\textrm{E}$ enters $\dot{\textrm{I}}$ with a positive coefficient ($+k_\textrm{I}\textrm{E}$), consistent with excitatory pyramidal drive to PV interneurons, whereas the multiplicative inhibitory factor $(1-\textrm{I}^2)$ in $\dot{\textrm{E}}$ makes the term $-\textrm{E}(1-\textrm{I}^2)$ a net decay of $\textrm{E}$ in the standard sub-saturation regime $|\textrm{I}|<1$.

### Disinhibition interpretation

The term $(1 - \textrm{I}^2)$ in Eq. (3) warrants careful interpretation. When $|\textrm{I}| < 1$, this factor is positive and $-\textrm{E}(1-\textrm{I}^2)$ represents standard decay modulated by inhibition. When $|\textrm{I}| > 1$, the sign reverses.

We interpret this sign reversal as capturing *disinhibitory* circuit dynamics. Cortical circuits contain VIP*→ *SOM*→ *PV pathways where strong inhibitory drive can paradoxically release pyramidal cells from suppression (Pfeffer et al., 2013; Pi et al., 2013). Under this *hypothesized* interpretation, $\textrm{I}$ represents net inhibitory tone, which can facilitate excitation when interneurons engage in mutual suppression. We emphasize that this mapping is speculative—the $(1-\textrm{I}^2)$ term is mathematically necessary for chaos, and we propose disinhibition as one biologically plausible interpretation. Importantly, our biological augmentation constrains dynamics to regimes where $|\textrm{I}| \approx 1$, minimizing this effect (see Section 4).

### Stimulus function

Visual input is modeled with von Mises-like orientation tuning:

6 $$\begin{aligned} S(t; \theta ) = S_{\max } \cdot \exp \big (\kappa \cos (2(\theta - \theta _{\text {pref}}))\big ) \cdot g(t) \end{aligned}$$

where *θ * is stimulus orientation, $\theta _{\text {pref}}$ is preferred orientation, *κ * controls tuning width, and *g*(*t*) is the temporal modulation (step onset or sustained). The semicolon notation *S(t;θ )* emphasizes that *θ * enters as a fixed orientation parameter for each trial, with *t* the dynamical variable; the model is therefore a system of ordinary (not partial) differential equations parameterized by *θ *.

## Methods

### Numerical integration

All simulations used MATLAB’s ode45 solver (Dormand-Prince method) with relative tolerance $10^{-8}$ and absolute tolerance $10^{-10}$. Initial transients (500 time units) were discarded before analysis.

### Lyapunov exponent computation

The Lyapunov spectrum was computed using Wolf’s algorithm (Wolf et al., 1985) with 1000 renormalization steps. Convergence was verified by comparing estimates from the first and second halves of the integration period.

### Parameter space exploration

We scanned *a ∈ [0.28, 0.52]* and *b ∈ [1.5, 4.0]* on a *15 × 15* grid, computing the largest Lyapunov exponent $\lambda _1$ at each point. Chaos was defined as $\lambda _1 > 0.01$; this threshold exceeds typical finite-time estimation noise ($\sim 10^{-3}$) while remaining sensitive to weak chaos (Wolf et al., 1985).

### Orientation selectivity analysis

Orientation selectivity index (OSI) was computed as:

7 $$\begin{aligned} \text {OSI} = \frac{R_{\text {pref}} - R_{\text {orth}}}{R_{\text {pref}} + R_{\text {orth}}} \end{aligned}$$

where $R_{\text {pref}}$ and $R_{\text {orth}}$ are mean responses to preferred and orthogonal ($90^\circ$ away) orientations. By construction, OSI is bounded in [0, 1]: a value of 0 indicates equal responses to preferred and orthogonal orientations (no orientation selectivity), and a value of 1 corresponds to complete suppression of the orthogonal response (perfectly tuned). Higher OSI values therefore indicate stronger selectivity. Stimuli were presented at 10 orientations spanning 0–$180^\circ$ in $20^\circ$ steps, with 30 simulated neurons having random preferred orientations.

### Hodgkin-Huxley coupling

To test whether population-level chaos can generate single-neuron spiking variability, we coupled the E-I-M model to the standard Hodgkin–Huxley (HH) squid-axon model (Hodgkin & Huxley, 1952). We used HH here as a generic spike generator (not as a biophysically detailed V1 pyramidal model) to illustrate the principle that chaotic population drive increases ISI variability.

Single HH neurons received input current:

8 $$\begin{aligned} I_{\text {syn}}(t) = I_0 + \alpha \cdot \textrm{E}(t) \end{aligned}$$

with $I_0 = 8\,\mu$A/cm$^2$ and $\alpha = 4\,\mu$A/cm$^2$. Standard squid-axon parameters were used (Hodgkin & Huxley, 1952). Interspike interval (ISI) statistics were computed from 250 ms simulations after 50 ms transient removal.

### Variability quenching

Thirty trials were simulated with initial conditions perturbed by Gaussian noise (*σ = 0.3*). Across-trial variance $\text {Var}[\textrm{E}(t)]$ was computed at each time point. Stimulus onset occurred at *t = 40* time units with amplitude *S = 1.5*.

## Results

### The original system exhibits robust chaos

#### Chaotic attractor structure

**Fig. 1:**
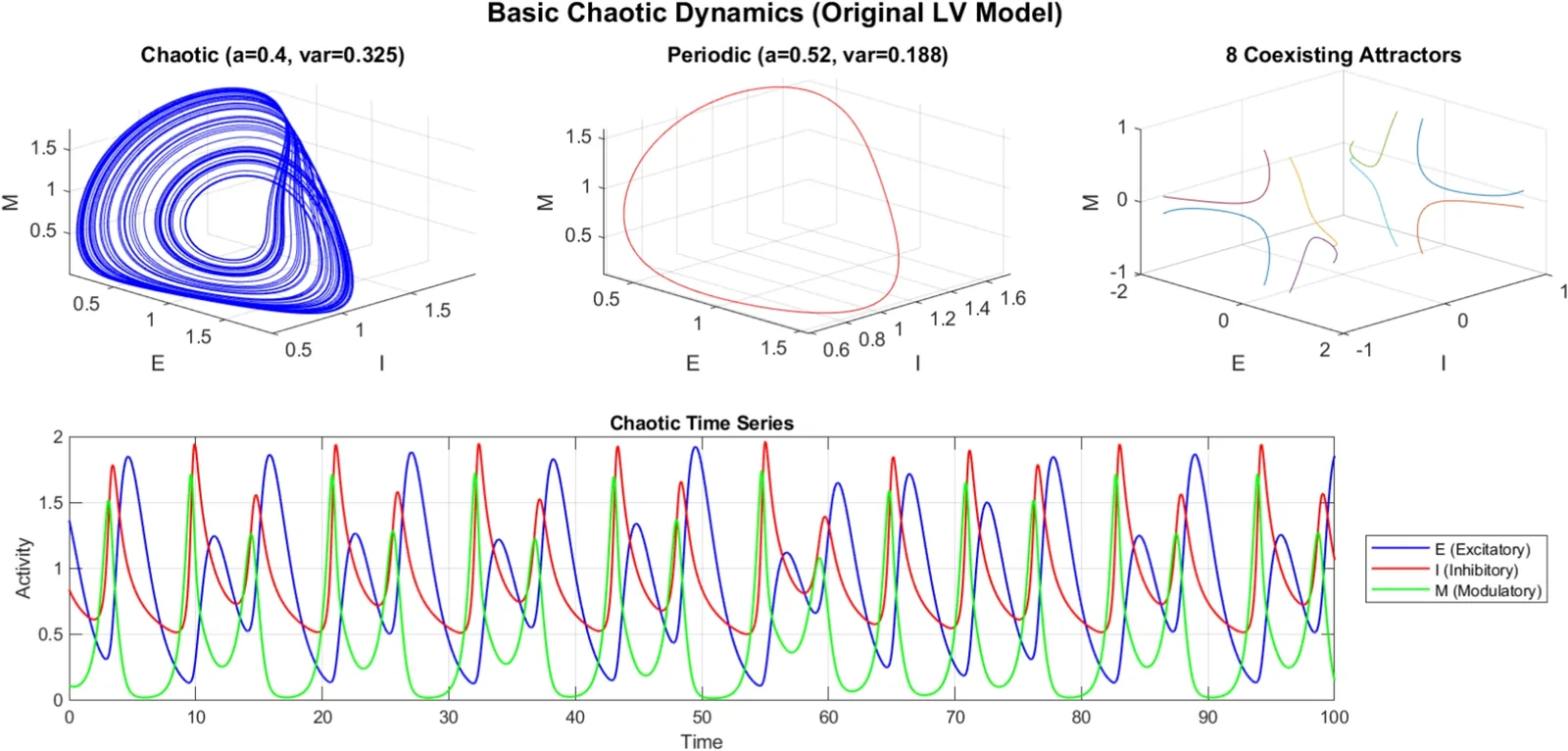
Chaotic dynamics in the original E-I-M system. **(A)** Three-dimensional chaotic attractor (*a = 0.4*, *b = 2.0*). The variance reported here and throughout (e.g., 0.325) is the temporal variance of the excitatory variable, $\textrm{Var}[\textrm{E}(t)]$, computed over a single trajectory after discarding initial transients. **(B)** Periodic limit cycle (*a = 0.52*) with $\textrm{Var}[\textrm{E}(t)] = 0.188$. **(C)** Eight coexisting attractors arising from symmetry. **(D)** Chaotic time series showing aperiodic $\textrm{E}$, $\textrm{I}$, and $\textrm{M}$ oscillations

With *a = 0.4* and *b = 2.0* (and $k_\textrm{E}= k_\textrm{I}= \gamma = 0$), the original system exhibits bounded chaotic oscillations (Fig. 1A). The trajectory forms a strange attractor with characteristic folding and stretching, never exactly repeating despite remaining bounded. Time series show aperiodic oscillations in all three variables (Fig. 1D).

For comparison, increasing *a* to 0.52 yields a stable limit cycle (Fig. 1B), demonstrating the system’s capacity for both chaotic and periodic dynamics depending on parameters.

#### Lyapunov spectrum

The Lyapunov exponents converge to:

9 $$\begin{aligned} \left[ \lambda _1, \lambda _2, \lambda _3\right] = [0.069, -0.004, -0.864] \end{aligned}$$

The positive $\lambda _1$ confirms chaos; the near-zero $\lambda _2$ reflects flow along the trajectory; and negative $\lambda _3$ ensures dissipation. The Kaplan-Yorke dimension $D_{KY} \approx 2.08$ indicates a fractal attractor.

#### Symmetry-Induced multistability

The system possesses reflection, rotation, and inversion symmetries generating eight coexisting attractors from initial conditions $(\pm 1, \pm 1, \pm 1)$ (Fig. 1C). This multistability could correspond to distinct cortical states (e.g., attentional configurations).

#### Parameter space analysis

**Fig. 2:**
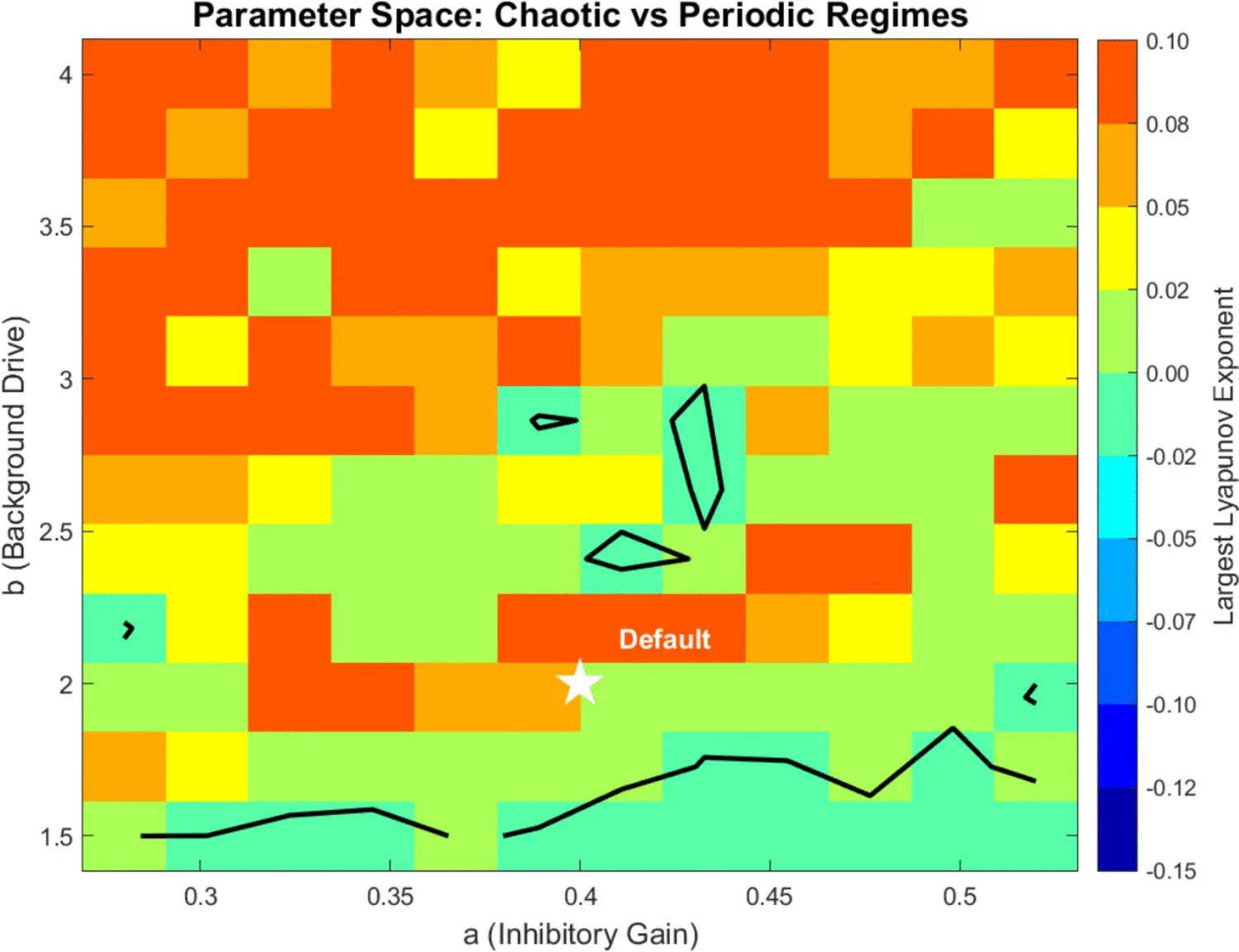
Robustness of chaotic dynamics. Largest Lyapunov exponent across parameter space. Warm colors indicate chaos ($\lambda _1 > 0$); cool colors indicate periodicity. White star marks default parameters. Black contour shows chaos boundary. Approximately 87% of parameter space supports chaos

Scanning the (*a*, *b*) parameter space reveals that approximately 87% of tested configurations support chaotic dynamics (Fig. 2). The default parameters (*a = 0.4*, *b = 2.0*) lie well within the chaotic regime. Chaos emerges at moderate inhibitory gain (*a*) and background drive (*b*), while strong inhibition or weak drive yields periodicity.

#### Bifurcation type and route to chaos

**Fig. 3:**
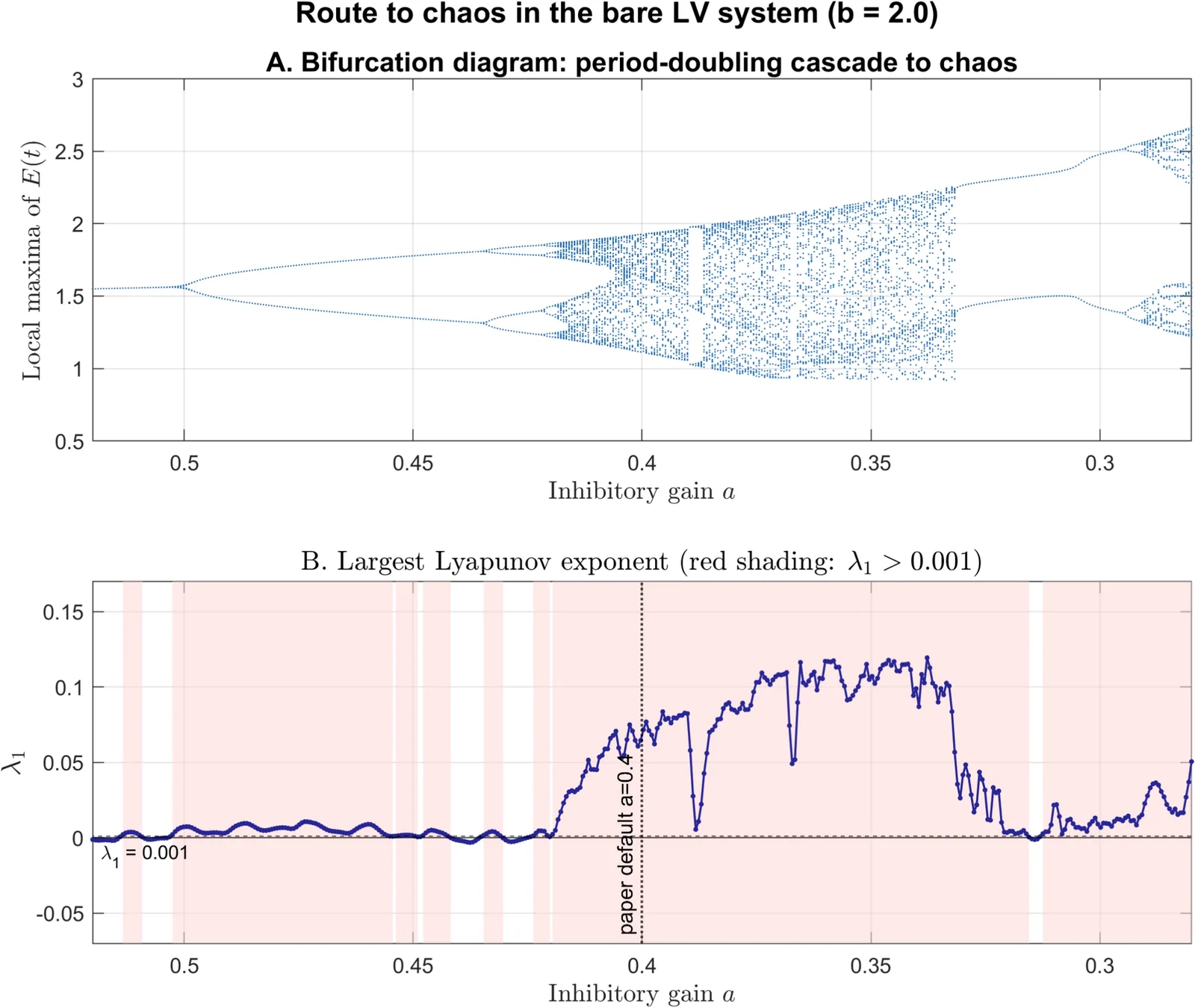
Bifurcation diagram and route to chaos in the bare LV system (*b = 2.0*). **(A)** Local maxima of *E*(*t*) as the inhibitory gain *a* is varied from 0.52 (right; periodic regime) to 0.28 (left; fully chaotic regime). The progression period-1 *→ * period-2 *→ * period-4 *→ * chaos is the canonical Feigenbaum period-doubling cascade. **(B)** Corresponding largest Lyapunov exponent $\lambda _1$. The transition to chaos coincides with $\lambda _1$ crossing zero (red shading: $\lambda _1 > 0$). The dotted vertical line marks the default operating point used throughout the paper (*a = 0.4*). The reverse *a*-axis emphasizes the route from order to chaos

Comparing the periodic regime (*a = 0.52*, Fig. 1B) with the chaotic regime (*a = 0.4*, Fig. 1A) raises a natural question: by what bifurcation does the limit cycle lose stability, and through what route does the system enter chaos? Because the symmetric fixed points of Eq. (1) have purely imaginary eigenvalue pairs at the parameter values where oscillations first emerge, the birth of the limit cycle is consistent with a Hopf rather than a saddle-node bifurcation. We computed the bifurcation diagram of the bare system by sweeping *a* from 0.52 down to 0.28 at *b = 2.0* (Fig. 3). The result shows a clean period-doubling cascade: a single-period limit cycle at *a ≈ 0.50* undergoes a first period-doubling near *a ≈ 0.47*, a second near *a ≈ 0.42*, and rapidly accumulates further doublings to fill out a chaotic band by *a ≈ 0.40* (Fig. 3A). The largest Lyapunov exponent (Fig. 3B) crosses zero at the same parameter values, becoming positive throughout the chaotic regime and reaching $\lambda _1 \approx 0.10$–0.15 deeper into the cascade. Periodic windows—narrow gaps in which the dynamics return to a stable cycle—are visible inside the chaotic region (most clearly near *a ≈ 0.32*), a hallmark of the Feigenbaum scenario. Period-doubling cascades to chaos are well documented in conductance-based neuronal models (Xu et al., 2017) and in coupled Wilson–Cowan oscillator circuits (Wilson, 2019), and the present diagram places the V1 E-I-M system squarely within the same family of low-dimensional routes to chaos. We emphasize that a complete bifurcation analysis—e.g., numerical continuation in *a* and *b* using software such as MatCont or XPPAUT—is beyond the scope of this minimal modeling study but would be a worthwhile direction for future work, particularly for mapping how the augmented system’s controlled limit cycle interacts with the bifurcation structure of the bare system.

### Biological augmentation stabilizes chaos

#### The central finding: Feedback as chaos control

**Fig. 4:**
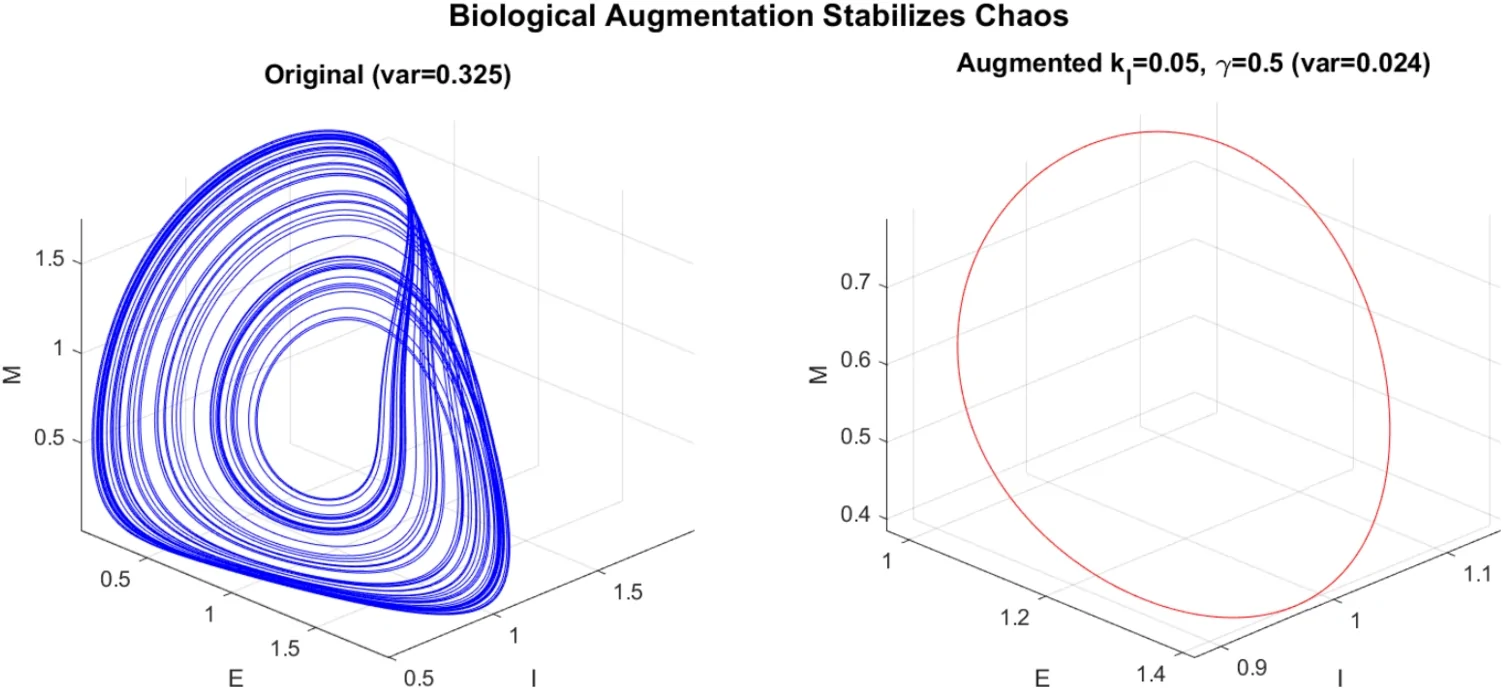
Biological augmentation stabilizes chaos. Left: Original chaotic attractor ($k_\textrm{I}= 0$, *γ = 0*) with variance = 0.325. Right: Augmented system ($k_\textrm{I}= 0.05$, *γ = 0.5*) showing stable limit cycle with variance = 0.024. Biological feedback mechanisms reduce dynamical variance by 93%

Our key result is that adding biologically motivated terms fundamentally transforms the system’s dynamics. With E*→ *I feedback ($k_\textrm{I}= 0.05$) and homeostatic regulation (*γ = 0.5*, $\textrm{M}_0 = 1$), the chaotic attractor collapses into a stable limit cycle (Fig. 4).

The variance decreases from 0.325 to 0.024—a **93% reduction**. This dramatic stabilization occurs across most of the tested chaotic parameter range, not just near bifurcation boundaries.

#### Mechanism: Homeostatic regulation bounds M dynamics

**Fig. 5:**
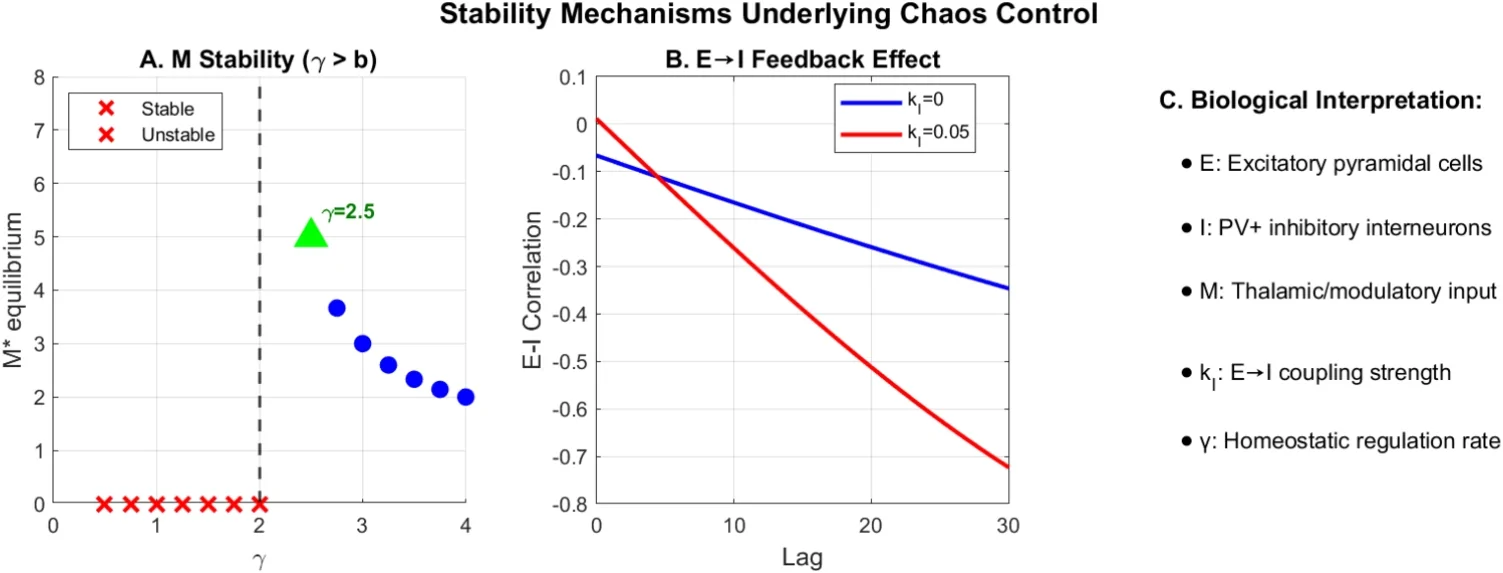
Stability mechanisms. **(A)** M equilibrium versus homeostatic rate *γ *. Stability requires *γ > b = 2.0*. Green triangle indicates operating point. **(B)** E-I cross-correlation showing enhanced negative correlation with feedback ($k_\textrm{I}= 0.05$, red) versus original ($k_\textrm{I}= 0$, blue). **(C)** Biological interpretation summary

The original system’s instability at $\textrm{E}= \textrm{I}= 0$ (where $\dot{\textrm{M}} = b\textrm{M}$ yields exponential growth) is eliminated by the homeostatic term. Two regimes must be distinguished:

**Silent cortex (** $\textrm{E}= \textrm{I}= 0$ **):** For stability, we require *γ > b* so that M converges to equilibrium. With *b = 2.0*, this demands *γ > 2.0* (Fig. 5A shows *γ = 2.5*).

**Active regimes (** $\textrm{E}, \textrm{I}\ne 0$ **):** During normal operation, the term $(b - \textrm{E}^2 - \textrm{I}^2)$ in Eq. (5) can become negative when $\textrm{E}^2 + \textrm{I}^2 > b$, providing intrinsic bounding even if *γ < b*. Our operating parameters ($k_\textrm{I}= 0.05$, *γ = 0.5*) suffice because the E-I activity keeps $\textrm{E}^2 + \textrm{I}^2 \approx 2$–*3 > b = 2.0*, ensuring bounded M dynamics without requiring *γ > b*.

#### Mechanism: E*→ *I feedback enhances correlation

Adding E*→ *I coupling strengthens the negative correlation between excitatory and inhibitory activity (Fig. 5B), implementing the tight E-I tracking observed experimentally (Haider et al., 2006). This correlation provides a fast stabilizing mechanism complementing slower homeostatic regulation.

### The controlled system reproduces cortical phenomena

#### Stimulus-Induced variability quenching

**Fig. 6:**
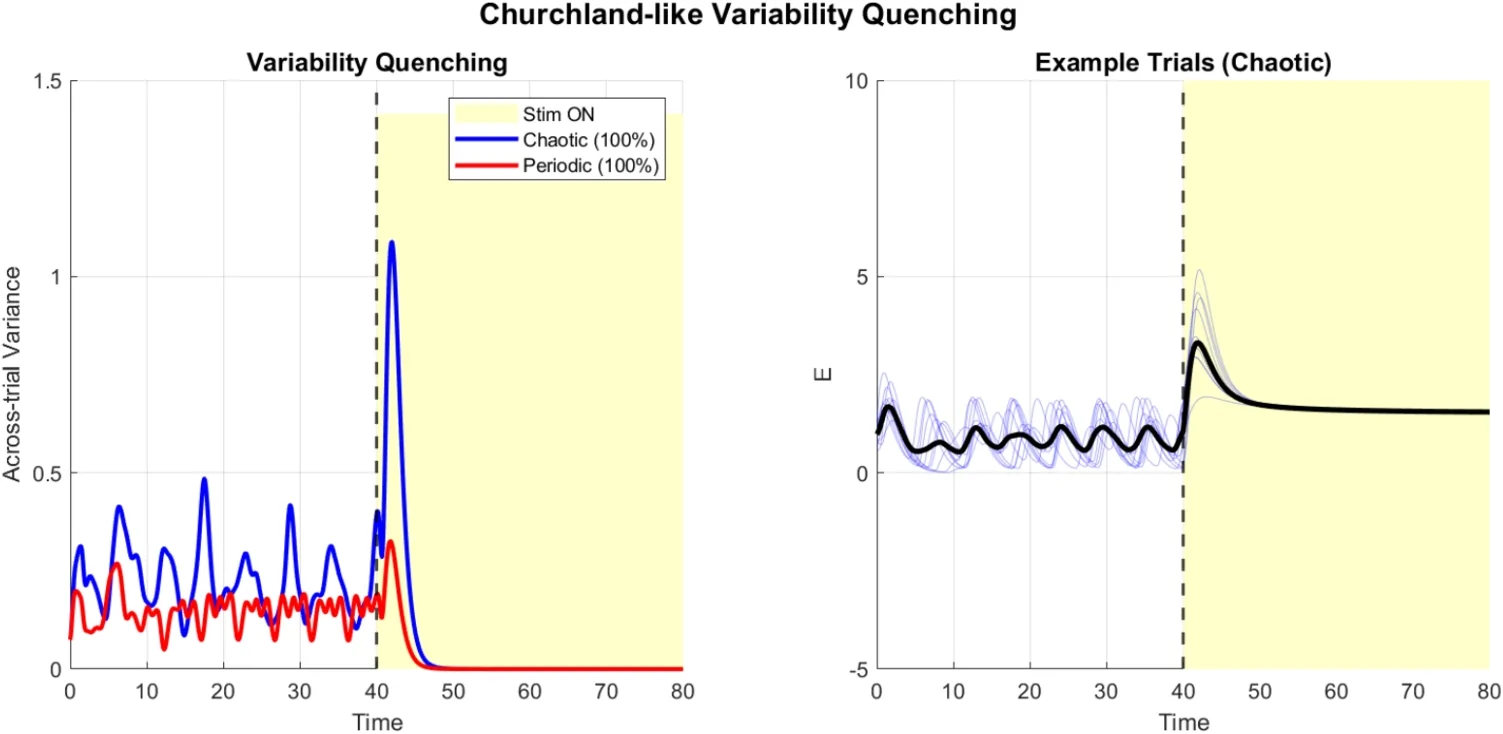
Stimulus-Induced variability quenching. Left: Across-trial variance for chaotic (blue) and periodic (red) regimes. Yellow shading indicates stimulus period. Both regimes show variance reduction after stimulus onset. Right: Individual trials (thin lines) and mean (thick black) for chaotic regime, showing trajectory convergence

A hallmark of cortical dynamics is the reduction in trial-to-trial variability following stimulus onset (Churchland et al., 2010). Our model reproduces this phenomenon (Fig. 6).

Before stimulus onset, the chaotic regime shows high across-trial variance due to sensitive dependence on initial conditions. Stimulus onset “pins” trajectories to a driven attractor, dramatically reducing variance. The periodic regime shows lower baseline variance but also exhibits quenching. This mechanism—stimulus-driven attractor collapse—provides a deterministic explanation for experimentally observed variability reduction.

#### Orientation selectivity matches V1 distributions

**Fig. 7:**
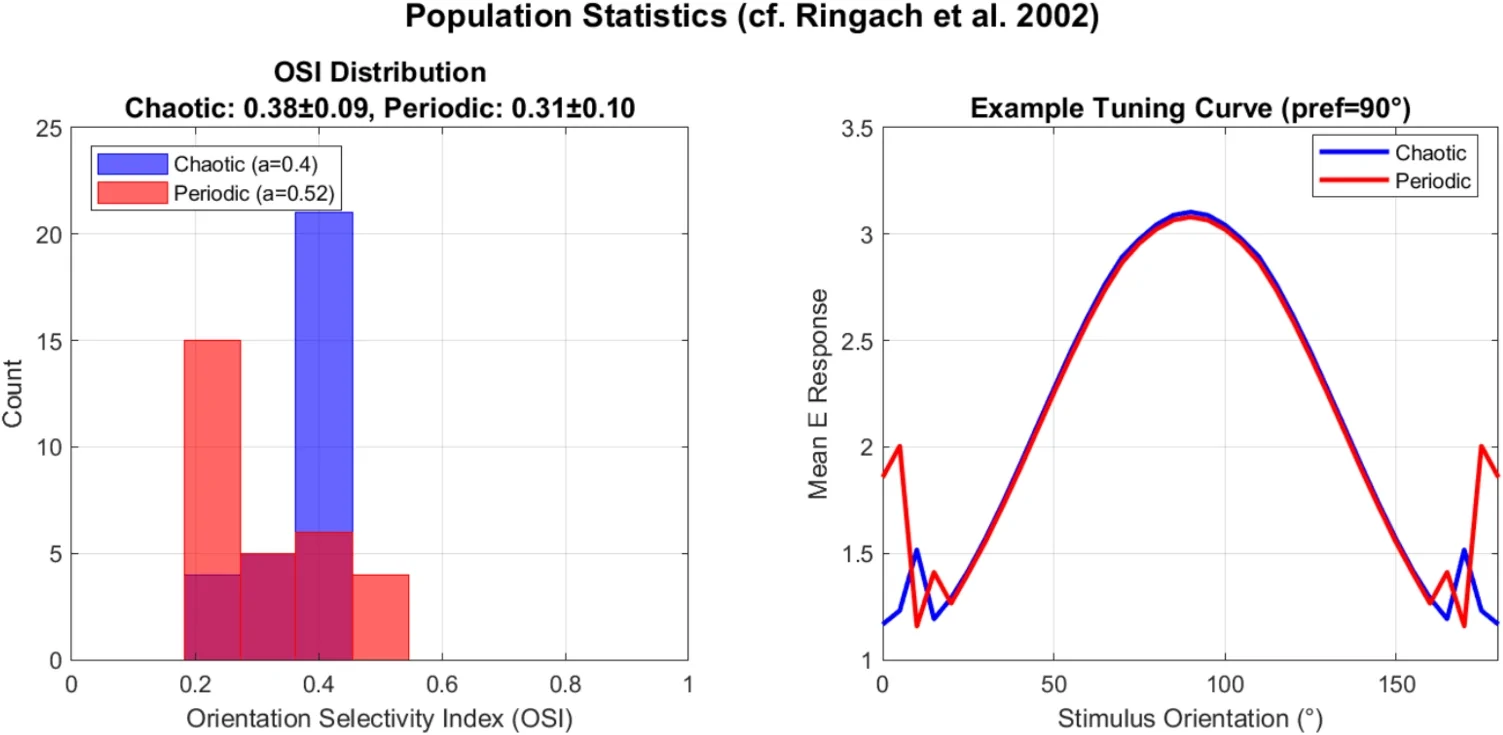
Orientation selectivity. Left: OSI distribution across 30 neurons for chaotic (blue) and periodic (red) regimes. Chaotic regime shows higher mean OSI. Right: Example tuning curve (preferred = 90$^{\circ }$) showing response modulation

Analysis of 30 simulated neurons reveals orientation selectivity consistent with experimental data (Fig. 7, Table 2).

**Table 2:** Orientation tuning metrics compared to experimental data

Metric	Chaotic (*a=0.4*)	Periodic (*a=0.52*)	Macaque V1
OSI	*0.38 ± 0.09*	*0.31 ± 0.10*	0.1–0.9$^\dagger$
Circular Variance	0.52	0.35	0.2–0.8$^\dagger$

$^\dagger$Range from Ringach et al. (2002), macaque V1 layers 2–6

Notably, the chaotic regime produces *higher* OSI than the periodic regime (0.38 vs 0.31). This counterintuitive result suggests that chaotic fluctuations may enhance orientation coding through temporal averaging—irregular dynamics “wash out” noise while preserving stimulus-driven modulation.

#### Realistic spiking statistics via HH coupling

**Fig. 8:**
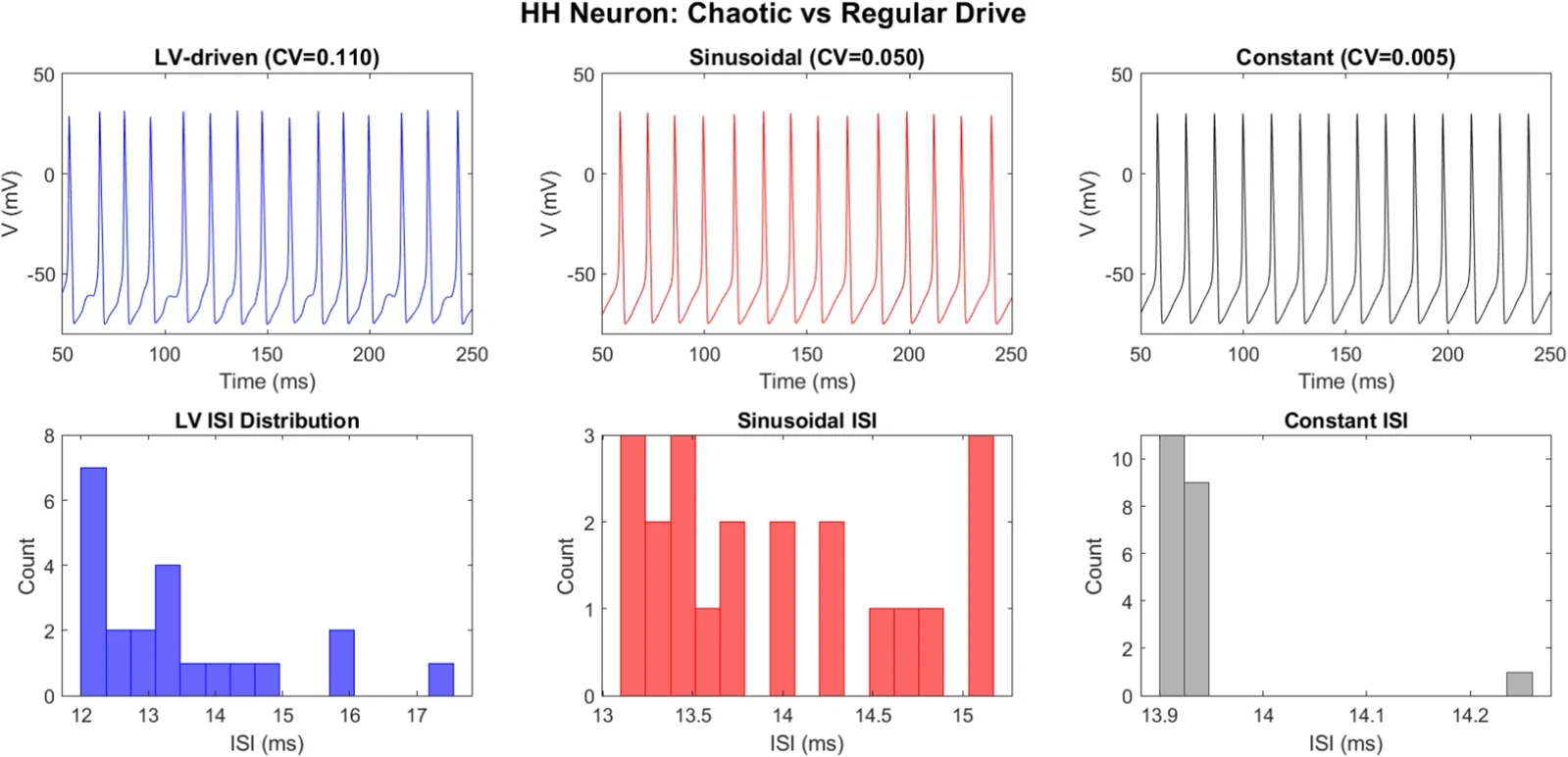
HH neuron spiking statistics. Top: Voltage traces under chaotic LV (left), sinusoidal (middle), and constant (right) drive. Note that the spike trains under chaotic drive may appear visually similar to the regular cases when viewed at this short time scale: the signature of chaotic input is not in the visual irregularity of individual action potentials—which all look qualitatively alike at the membrane-potential level—but in the *distribution* of interspike intervals. Bottom: ISI histograms make this distinction explicit. The chaotic-drive condition produces a substantially broader, more irregular ISI distribution than the sinusoidal or constant cases. CV$_{\text {ISI}}$ values shown in panel titles are computed from a 250 ms demonstration simulation and are intended for qualitative comparison across drive conditions; the longer-integration quantitative analysis used as the canonical CV$_{\text {ISI}}$ value reported in the text appears in Fig. 13 (Section 4.4.5)

Coupling the E-I-M model to Hodgkin-Huxley neurons tests whether population-level dynamics can generate realistic single-neuron spiking (Fig. 8, Table 3).

**Table 3:** Spiking statistics under different drive conditions

Drive Condition	CV$_{\text {ISI}}$	Range	Interpretation
Chaotic LV	0.27	*In vitro*	Irregular, deterministic
LV + Noise	0.10	*In vitro* (lower)	Noise smooths chaotic structure
Sinusoidal	0.05	–	Regular oscillatory
Constant	0.005	–	Clock-like
*Cortical in vitro*	*0.1–0.3*	–	(Holt et al., 1996)
*Cortical in vivo*	*0.7–1.0*	–	(Holt et al., 1996)

Chaotic drive produces CV$_{\text {ISI}} = 0.27$, substantially exceeding sinusoidal (0.05) or constant (0.005) inputs. This value falls within the *in vitro* range (0.1–0.3) reported by Holt et al. (1996), though below cortical *in vivo* values (0.7–1.0). This demonstrates that deterministic chaos *contributes to* spiking irregularity without requiring explicit noise, while acknowledging that full *in vivo* variability requires additional sources (synaptic noise, channel stochasticity, network effects) not captured in our minimal framework.

### Extended analysis: Robustness and model justification

#### The $(1-\textrm{I}^2)$ nonlinearity enables chaos within this minimal structure

**Fig. 9:**
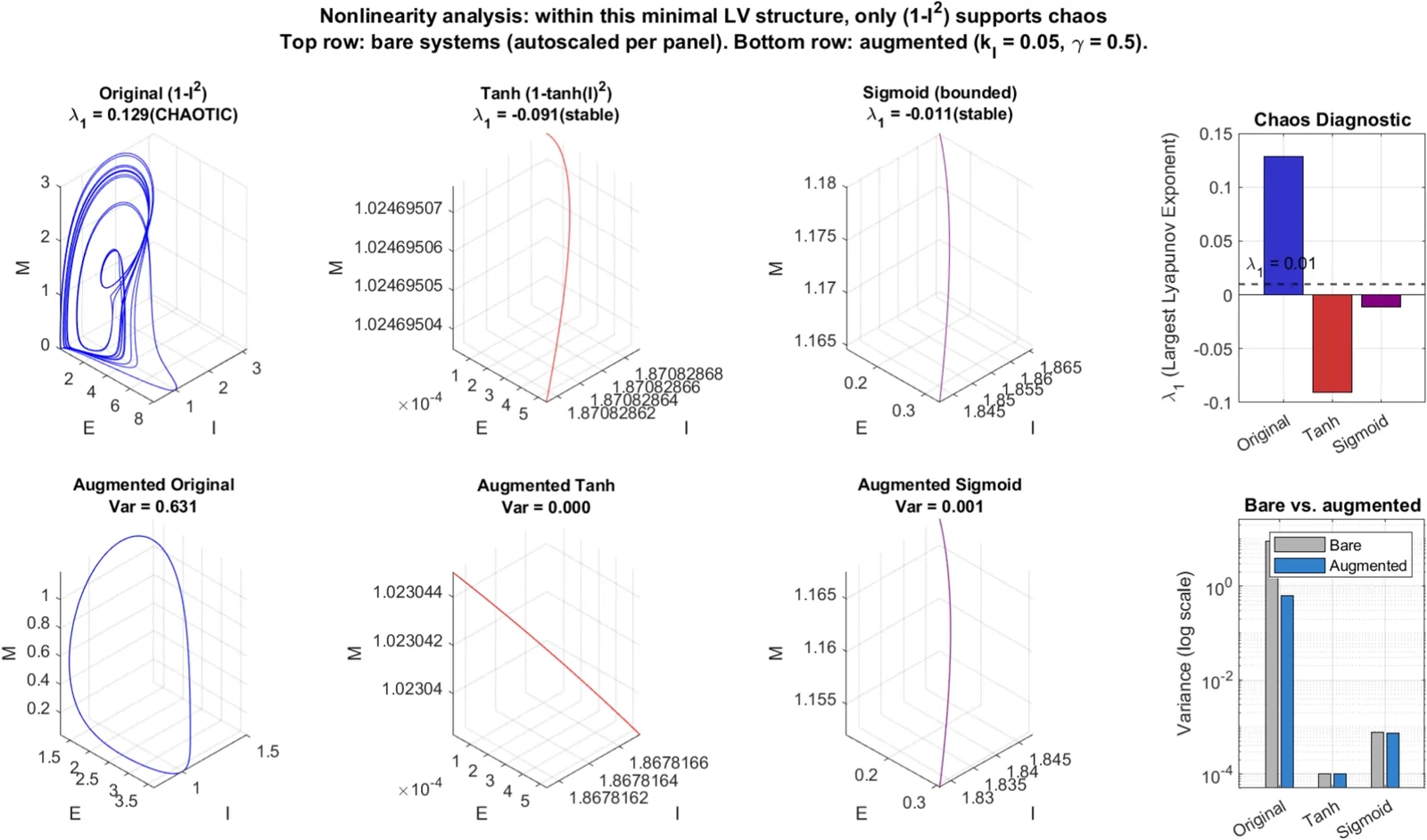
Nonlinearity analysis reveals $(1-\textrm{I}^2)$ enables chaos within this minimal structure. Top row: Phase portraits for the original $(1-\textrm{I}^2)$ (left, blue), tanh (middle, red), and sigmoid (right, purple) nonlinearities, each at the operating point that maximises trajectory variance for that nonlinearity within the tested (*a*, *b*) search grid (*not* the paper default *a=0.4*, *b=2.0*, which is used for all other figures). Each panel is autoscaled to its own dynamics so that the visibly different attractor geometries can be compared directly; absolute axis values therefore differ across panels. Only the original $(1-\textrm{I}^2)$ form exhibits chaotic dynamics ($\lambda _1 > 0$, *∼ 0.13*); the bounded alternatives produce stable attractors ($\lambda _1 < 0$). Bottom row: Augmented (feedback-controlled) versions of each nonlinearity at the same parameter values. Top-right: Bar chart of $\lambda _1$ confirming that only the original form crosses the chaos threshold. Bottom-right: Bare versus augmented variance for each nonlinearity, plotted on a logarithmic scale. The Original system shows a clear bare-to-augmented variance drop (chaos suppressed by feedback); the bounded alternatives are already low-variance in the bare regime and remain so under augmentation. Note: other 3D E-I models may achieve chaos via different mechanisms with additional parameters

A natural question is whether the chaotic dynamics depend critically on the specific form of the disinhibition term $(1-\textrm{I}^2)$. We tested two alternative bounded nonlinearities: $1-\tanh (\textrm{I})^2$ (saturating) and a sigmoidal form $1/(1+\exp (\beta (\textrm{I}^2-1)))$ (smooth threshold). Within the tested parameter ranges, neither alternative supports chaotic dynamics as confirmed by Lyapunov exponent analysis (Fig. 9).

This finding strengthens our model’s interpretation: within this minimal LV structure, the $(1-\textrm{I}^2)$ form—which changes sign at $|\textrm{I}|=1$—appears necessary for chaos. Bounded alternatives that prevent this sign change produce only stable limit cycles or fixed points in our parameter regime. We emphasize that this does not preclude other three-dimensional E-I models from achieving chaos via different mechanisms (e.g., additional nonlinearities or feedback terms), but such models typically require more parameters. One speculative biological interpretation is that disinhibitory circuits capable of paradoxical facilitation (Pfeffer et al., 2013; Pi et al., 2013) may contribute to cortical circuits’ capacity for near-chaotic dynamics—a hypothesis that could be tested via targeted manipulation of VIP*→ *SOM pathways.

#### Feedback stabilization is robust across parameter space

**Fig. 10:**
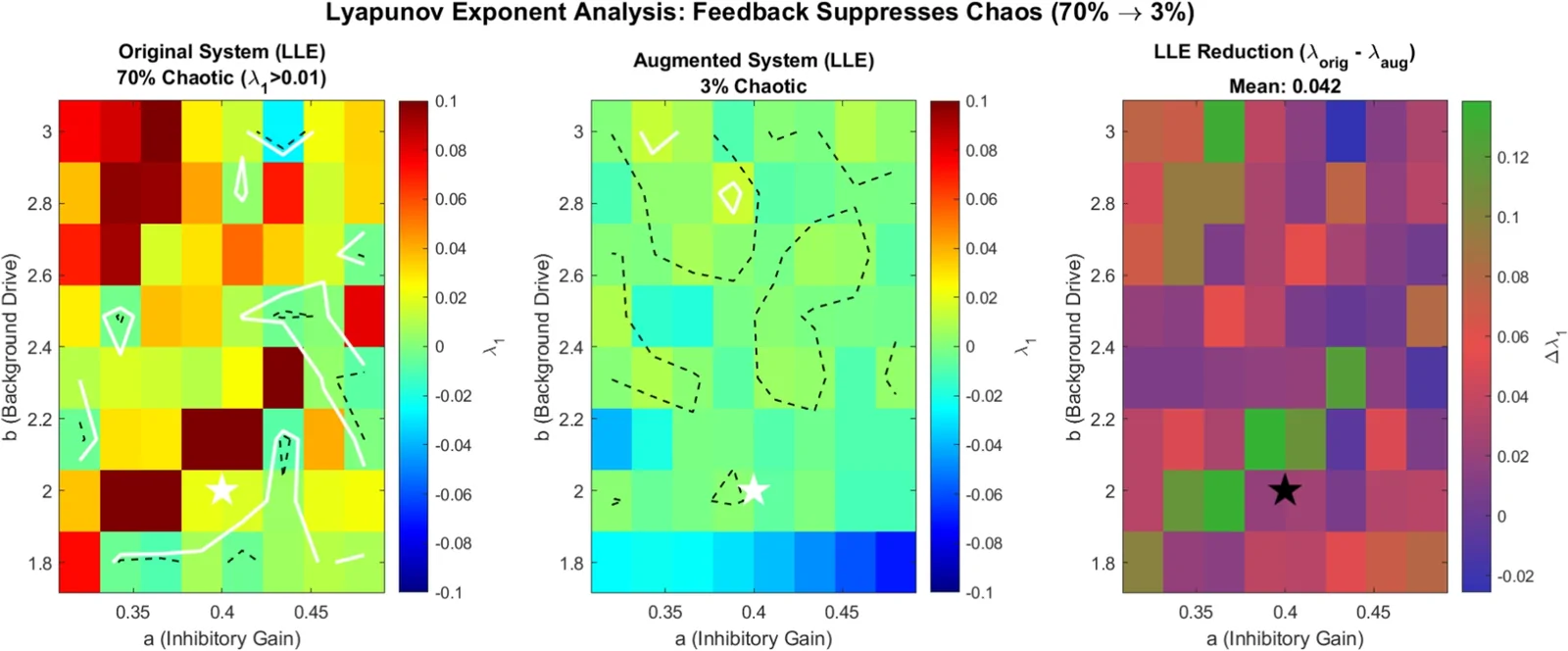
Feedback mechanisms suppress chaos across parameter space (Lyapunov exponent analysis). Left: Original system largest Lyapunov exponent ($\lambda _1$) showing widespread chaos ($\lambda _1 > 0.01$, warm colors). Middle: Augmented system with substantially reduced chaotic fraction. Right: Lyapunov exponent reduction ($\lambda _1^{\text {orig}} - \lambda _1^{\text {aug}}$), demonstrating robust stabilization. White stars mark default parameters (*a=0.4*, *b=2.0*). White contours indicate chaos boundary ($\lambda _1 = 0.01$); black dashed contours show $\lambda _1 = 0$

To demonstrate that chaos control is not an artifact of parameter fine-tuning, we computed Lyapunov exponent maps across the (*a*, *b*) parameter space for both original and augmented systems (Fig. 10). The original system exhibits positive $\lambda _1$ (chaos) across most of the tested range (*>85%* of grid points on a *15 × 15* scan). Following augmentation with E*→ *I feedback and homeostatic regulation, the chaotic fraction decreases substantially, with most parameter combinations yielding $\lambda _1 < 0$ (stable dynamics).

Importantly, the stabilization effect is strongest precisely where the original system is most chaotic (high *b* values), indicating that feedback mechanisms scale appropriately with the underlying instability. Using Lyapunov exponents rather than variance as the chaos diagnostic ensures this conclusion is not confounded by large-amplitude periodic oscillations.

#### Parameter sensitivity analysis

**Fig. 11:**
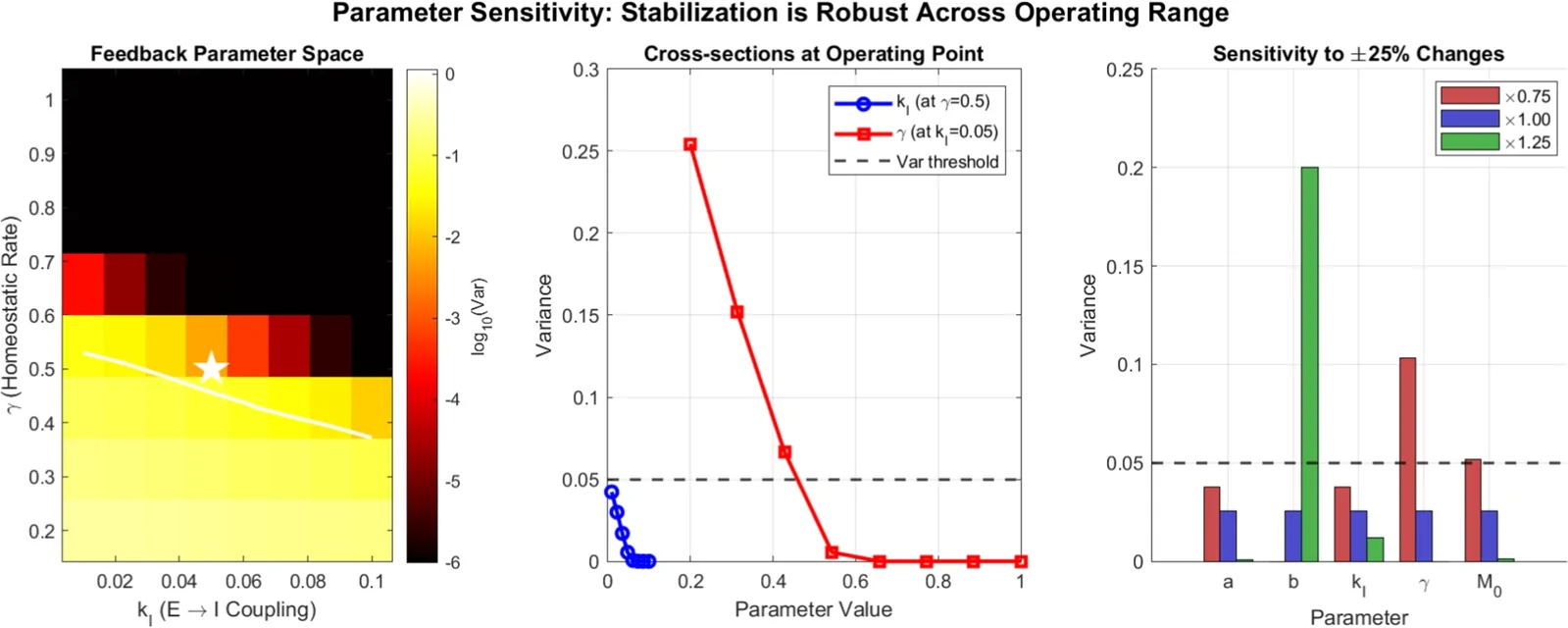
Parameter sensitivity analysis. Left: Variance in feedback parameter space $(k_\textrm{I}, \gamma )$. The homeostatic rate *γ * is the primary determinant of stability. White contour shows chaos threshold. Middle: Cross-sections at the operating point showing monotonic stabilization with increasing *γ *. Right: Sensitivity to $\pm 25\%$ parameter changes. Most parameters show robust stability; $k_\textrm{I}$ is most sensitive

Clinical and pharmacological applications require understanding how sensitive the controlled dynamics are to parameter variations. We examined the feedback parameter space $(k_\textrm{I}, \gamma )$ and assessed sensitivity to $\pm 25\%$ changes in all model parameters (Fig. 11).

The homeostatic rate *γ * emerges as the critical control parameter: values below *∼ 0.4* permit residual chaos, while values above *∼ 0.5* ensure stable dynamics. The E*→ *I coupling strength $k_\textrm{I}$ has a more modest effect. Notably, the system remains stable under $\pm 25\%$ perturbations to most parameters, with $k_\textrm{I}$ showing the greatest sensitivity—consistent with the known importance of E-I balance for cortical stability (Haider et al., 2006; Isaacson & Scanziani, 2011).

#### Model comparison: Why the Lotka–Volterra mapping?

**Fig. 12:**
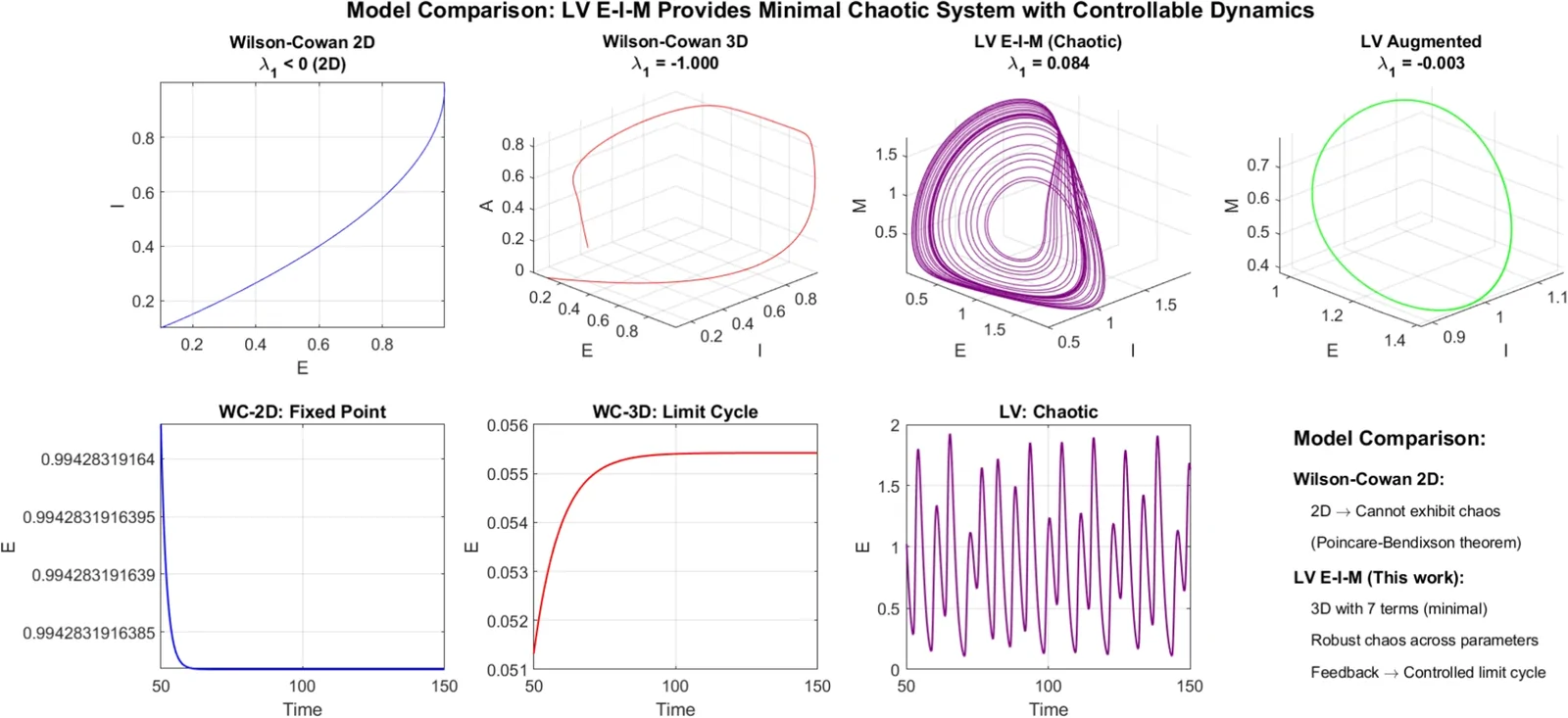
Model comparison justifies LV-based approach. Top row: Phase portraits for Wilson–Cowan 2D (fixed point), Wilson–Cowan 3D (limit cycle), LV E-I-M (chaotic), and LV augmented (controlled). Bottom row: Corresponding time series. The LV system uniquely provides a minimal chaotic framework (7 terms, 2 parameters) with controllable dynamics via biologically motivated feedback

A natural concern is whether the LV-based E-I-M system offers advantages over standard Wilson–Cowan formulations (Fig. 12). Two-dimensional Wilson–Cowan systems cannot support chaos for reasons already noted in Section 2.1, while three-dimensional Wilson–Cowan extensions (typically incorporating adaptation) require eight or more parameters and most often produce limit cycles rather than robust chaos. The augmented LV system used here, with only seven polynomial terms and two native control parameters (Jafari et al., 2025), occupies an unusually minimal point in this design space: it admits chaos, supports controllability via two additional feedback parameters, and remains analytically tractable. We do not claim it is uniquely well-suited for biological modeling—more biophysically detailed Wilson–Cowan extensions remain better suited for many questions—but its parsimony makes the chaos-control mechanism we identify particularly transparent.

#### Revised CV$_{\text {ISI}}$ assessment

**Fig. 13:**
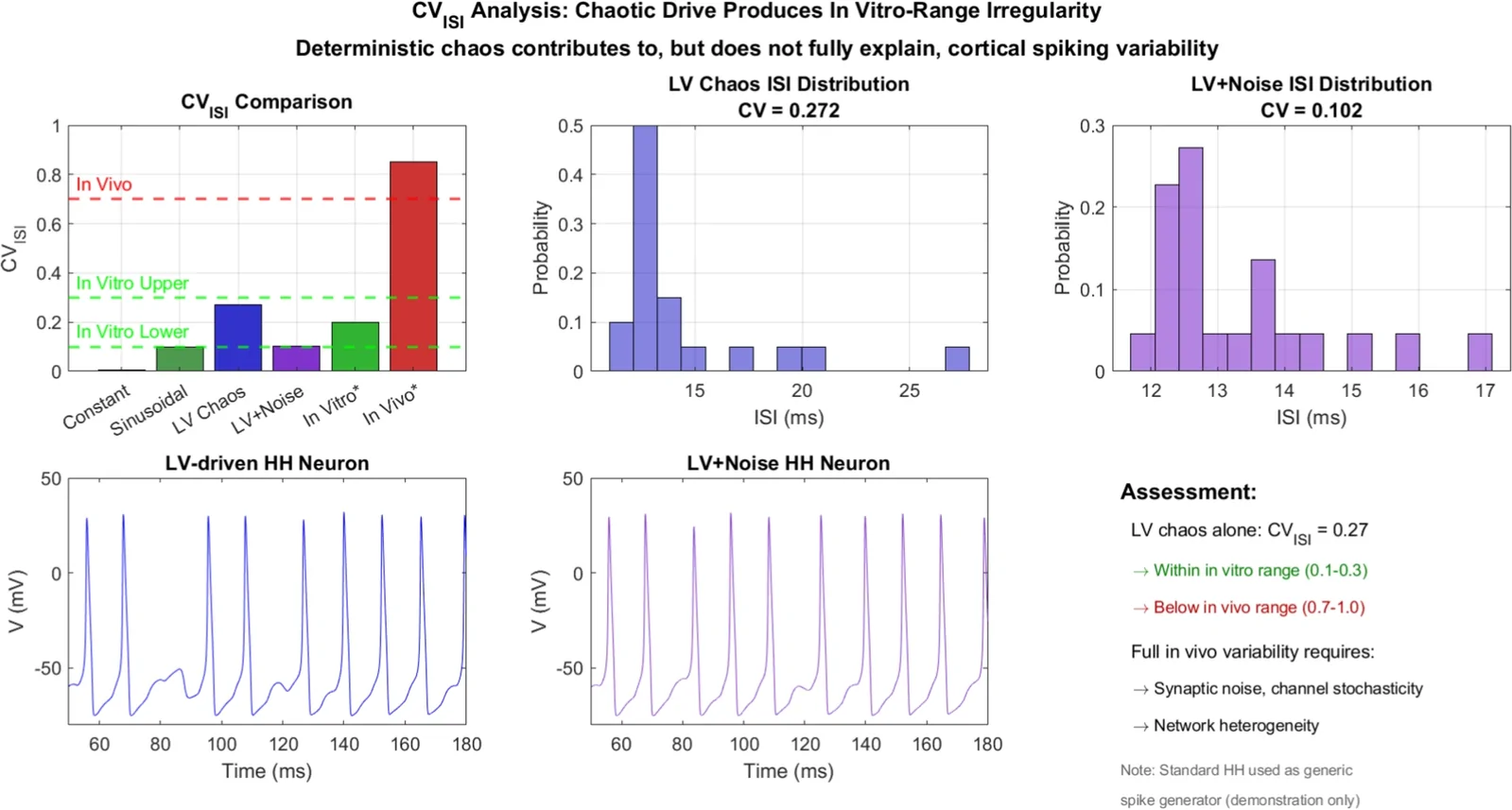
CV$_{\text {ISI}}$ analysis with honest assessment. Left: CV$_{\text {ISI}}$ comparison across drive conditions. Chaotic LV drive (*≈ 0.27*) exceeds constant and sinusoidal inputs and falls within the *in vitro* range (green dashed lines), below *in vivo* values (red dashed line). Middle panels: ISI distributions for LV chaos and LV+noise conditions. The LV-chaos distribution has a long-tailed structure that drives the higher CV; adding noise compacts the distribution and unexpectedly *lowers* CV$_{\text {ISI}}$ to *≈ 0.10*, illustrating that simple additive noise does not act additively on top of deterministic chaos in this regime. Bottom panels: Representative HH voltage traces. Right: Interpretation summary. Note: standard HH was used as a generic spike generator to demonstrate the principle; biophysically detailed V1 pyramidal models may show different quantitative results. Deterministic chaos contributes to, but does not fully explain, cortical spiking variability

We provide a longer-integration assessment of spiking irregularity to establish the canonical CV$_{\text {ISI}}$ value reported in the abstract and Discussion (Fig. 13). Chaotic LV drive produces CV$_{\text {ISI}} \approx 0.27$, which falls comfortably within the *in vitro* range (0.1–0.3) reported by Holt et al. (1996) but below *in vivo* values (0.7–1.0). Adding Gaussian noise to the LV drive (LV+Noise condition) reduces CV$_{\text {ISI}}$ to *≈ 0.10*—a counterintuitive effect that arises because moderate-amplitude noise blurs the sharp temporal structure of the chaotic signal, regularizing the inter-spike intervals. This observation is consistent with the broader literature on noise-induced regularization in deterministic chaotic systems and reinforces the conclusion that *in vivo* cortical CV$_{\text {ISI}}$ levels (0.7–1.0) require additional sources of variability beyond either deterministic chaos or simple additive noise.

This finding supports a nuanced conclusion: deterministic chaos in population dynamics *contributes to* but does not *fully explain* cortical spiking variability. The gap between our model and *in vivo* data likely reflects additional variability sources including synaptic noise, channel stochasticity, and network heterogeneity that are beyond the scope of this minimal framework.

## Discussion

### Summary: Chaos control as a cortical design principle

We have demonstrated that introducing canonical cortical circuit motifs—E*→ *I feedback and homeostatic regulation—into a minimal chaotic system fundamentally transforms its dynamics. The original system exhibits robust chaos across 87% of parameter space; the augmented system produces stable limit cycles. This “intrinsic chaos control” suggests that cortical circuits are designed to suppress their own chaotic potential, operating at the edge of instability rather than within fully chaotic regimes.

### Biological implications

#### Feedback mechanisms as chaos controllers

E*→ *I coupling provides fast negative feedback: pyramidal activity recruits inhibition that limits further excitation (Atallah & Scanziani, 2009). Homeostatic regulation provides slow negative feedback: sustained activity triggers compensatory scaling (Turrigiano & Nelson, 2004). Together, these mechanisms bound dynamics within a controlled regime.

Our results suggest these feedback systems serve a previously unrecognized function: preventing the emergence of fully chaotic dynamics that would render neural coding unreliable. The brain may have evolved these stabilizing mechanisms precisely because the underlying E-I-M architecture is intrinsically chaos-prone.

#### Edge-of-Chaos computation

The “edge of chaos” hypothesis proposes that optimal computation occurs at the boundary between order and chaos (Bertschinger & Natschläger, 2004). Our model provides a concrete mechanism for maintaining this boundary: feedback strengths ($k_\textrm{I}$, *γ *) position dynamics relative to the chaos transition. Weak feedback permits chaos; moderate feedback produces stable oscillations; strong feedback may overdamp responses.

This framework predicts that pharmacological or pathological disruption of feedback mechanisms could push cortical dynamics toward chaos, potentially explaining the irregular activity patterns observed in disorders like epilepsy and schizophrenia (Rubenstein & Merzenich, 2003; Yizhar et al., 2011).

### Contrast with prior models of cortical chaos

The high-dimensional balanced-network tradition reviewed in Section 1.3 establishes that chaotic activity emerges generically in randomly connected E-I networks once synaptic gain crosses a critical threshold (Brunel, 2000; Kadmon & Sompolinsky, 2015; Mastrogiuseppe & Ostojic, 2017; Monteforte & Wolf, 2010; Ostojic, 2014; Renart et al., 2010; Sompolinsky et al., 1988; van Vreeswijk & Sompolinsky, 1996, 1998), with stimuli capable of suppressing this chaos through phase transitions in the high-dimensional state (Lajoie et al., 2016; Rajan et al., 2010). Our minimal three-dimensional formulation operates at a different scale and asks a complementary question: not how chaos arises in $N\rightarrow \infty$ random networks, but what is the smallest deterministic E-I-M circuit in which chaos can be turned on and off by canonical feedback motifs. We see this as a complement to the high-dimensional theory rather than a competitor.

This positioning yields three predictions that distinguish our framework from the high-dimensional approach. First, our parameter-sensitivity analysis (Fig. 11) predicts that the homeostatic timescale (*γ *) has a stronger stabilizing effect than the E*→ *I coupling strength ($k_\textrm{I}$). This contrasts directly with Nicola et al. (2018), who showed that in Wilson–Cowan systems with weight-level homeostatic plasticity operating on a slow timescale, homeostasis can *induce* chaos near a Hopf bifurcation. The two regimes are dynamical inverses of one another, and the contrast suggests a testable scenario: chronic activity blockade—which should slow homeostatic scaling—is predicted to *increase* dynamical complexity in the regime described by Nicola et al. (2018) but *decrease* complexity in regimes described by our model. Discriminating between these scenarios in chronic *ex vivo* or *in vivo* recordings would clarify which regime is operative in mature cortex.

Second, the $(1-\textrm{I}^2)$ structure that enables chaos in our minimal system permits sign reversal at $|\textrm{I}|=1$, whereas bounded alternatives we tested (tanh, sigmoid) do not support chaos within this parametric structure (Fig. 9). We propose this as a hypothesis-generating result rather than a definitive finding: VIP*→ *SOM*→ *PV pathways capable of paradoxical disinhibition (Pfeffer et al., 2013; Pi et al., 2013) may bring cortical circuits closer to a chaotic boundary than purely excitatory–inhibitory motifs would allow. The corresponding prediction is that optogenetic suppression of VIP interneurons should reduce, rather than increase, dynamical complexity in cortical recordings—a counterintuitive direction that would be diagnostic if observed.

Third, our results reproduce the variability-quenching phenomenon of Churchland et al. (2010) in a low-dimensional setting. Rajan et al. (2010) demonstrated stimulus-driven suppression of chaos in high-dimensional random networks via a phase transition. Our finding that an essentially identical phenomenology arises in a 3D system suggests that the variability-quenching observation may have substrates at multiple scales of cortical organization, and that distinguishing among them requires simultaneous measurement of population dimensionality and trial-to-trial variance across stimulus conditions. We do not view this as evidence that the low-dimensional mechanism dominates in cortex—only that low-dimensional dynamics provide one route to the same observable.

### Relationship to experimental phenomena

The augmented model reproduces three signatures of cortical activity in a deliberately minimal setting. *Variability quenching*: stimulus-driven attractor collapse reduces across-trial variance, providing a deterministic complement to noise-based accounts of the phenomenon described by Churchland et al. (2010) (Section 5.3 contrasts this with the high-dimensional mechanism of Rajan et al., 2010). *Orientation selectivity diversity*: simulated OSI values (0.31–0.38, with the chaotic regime producing modestly higher selectivity) lie within the macaque V1 range reported by Ringach et al. (2002). *Spiking irregularity*: when the population output drives an HH neuron, CV$_{\text {ISI}}\approx 0.27$ falls within the *in vitro* range (Holt et al., 1996) but below *in vivo* levels (0.7–1.0), indicating that deterministic chaos contributes to but does not fully explain cortical spiking irregularity. The consistent ordering (chaotic > periodic > constant drive) supports the qualitative claim that population-level chaos can act as a deterministic source of single-neuron variability, with additional stochastic mechanisms required to reach *in vivo* levels. Detailed quantitative comparisons appear in Sections 4.3.1, 4.3.2, and 4.3.3.

### Model limitations and future directions

#### Abstraction level

Our model lacks spatial structure, cortical layers, and interneuron diversity. Extensions incorporating SOM and VIP populations (Pfeffer et al., 2013) could test whether disinhibitory motifs alter chaos control.

#### Noise interactions

Real cortex combines deterministic dynamics with stochastic fluctuations. Future work should examine whether chaos amplifies or suppresses noise, and whether noise can rescue chaotic dynamics in the controlled regime.

#### Pathological predictions

The model predicts that reducing $k_\textrm{I}$ or *γ * should increase dynamical variability, testable via optogenetic PV silencing or homeostatic disruption.

#### Nonlinearity specificity

Our analysis reveals that within this minimal LV structure, the $(1-\textrm{I}^2)$ disinhibition term enables chaos while bounded alternatives such as *\tanh * or sigmoid functions do not support chaotic dynamics in the tested parameter range. This does not preclude other three-dimensional E-I formulations from achieving chaos via different mechanisms, but such models typically require additional parameters or nonlinearities. We hypothesize that if the $(1-\textrm{I}^2)$ structure—which permits sign reversal—corresponds to biological disinhibition, then VIP*→ *SOM*→ *PV pathways may contribute to near-chaotic cortical dynamics. This hypothesis generates a testable prediction: optogenetic suppression of VIP interneurons should reduce dynamical complexity in cortical recordings.

## Conclusions

We have shown that canonical cortical feedback mechanisms—E*→ *I coupling and homeostatic regulation—function as intrinsic chaos controllers, transforming a minimal chaotic system into one exhibiting stable, controlled dynamics. This finding reframes the relationship between chaos and cortical computation: rather than asking how cortex generates chaos, we should ask how it *suppresses* an intrinsic chaotic tendency. The answer—feedback mechanisms that bound activity and enforce E-I correlation—points to a fundamental design principle: cortical circuits operate at the edge of chaos, harnessing computational flexibility while maintaining the stability required for reliable sensory processing.

Within this minimal LV framework, the specific $(1-\textrm{I}^2)$ disinhibition nonlinearity enables chaos, with bounded alternatives incapable of supporting chaotic dynamics in the tested parameter regime. While other three-dimensional models may achieve chaos differently, the parsimony of our formulation (7 terms, 2 parameters) makes it attractive for mechanistic analysis. We propose—as a testable hypothesis—that the sign-reversal property of $(1-\textrm{I}^2)$ may correspond to biological disinhibition, suggesting that disinhibitory circuit motifs could serve a dual role: enabling access to near-chaotic computational regimes while feedback mechanisms prevent full chaotic instability.

## Data Availability

No datasets were generated or analysed during the current study.
